# Leukocyte mitochondrial DNA copy number and cardiovascular disease: A systematic review and meta-analysis of cohort studies

**DOI:** 10.1016/j.isci.2024.110522

**Published:** 2024-07-17

**Authors:** Xinying Li, Xiaoning Liu, Xiaojuan Chen, Yanqi Wang, Shuning Wu, Fengjuan Li, Yuhao Su, Lifang Chen, Jian Xiao, Jianping Ma, Pei Qin

**Affiliations:** 1 Center for Clinical Epidemiology and Evidence-Based Medicine, Shenzhen Qianhai Shekou Free Trade Zone Hospital, Shenzhen, Guangdong, China; 2 School of Public Health, Shantou University, Shantou, Guangdong, China; 3 Department of Respiratory and Critical Care Medicine, The Second Affiliated Hospital of Zhengzhou University, Zhengzhou, China; 4 Department of Thyroid and Breast Surgery, Shenzhen Qianhai Shekou Free Trade Zone Hospital, Shenzhen, China; 5 Department of Cardiovascular Medicine, Shenzhen Qianhai Shekou Free Trade Zone Hospital, Shenzhen 518000, Guangdong, China

**Keywords:** Health sciences, Medicine, Medical specialty, Internal medicine, Cardiovascular medicine

## Abstract

Increasing cohort studies have examined the link between mitochondrial DNA copy number (mtDNA-CN) and cardiovascular disease (CVD), with inconsistent findings. We searched PubMed, EMBASE, and Web of Science up to July 11, 2023 and used a random-effects model to calculate summary hazard ratios (HRs) and 95% confidence intervals (CIs). This systematic review and meta-analysis included 8 articles encompassing 29 studies with 646,398 participants. Individuals with the lowest mtDNA-CN had a summary HR of 1.27 (95% CI 1.02–1.59) for CVD, 1.18 (95% CI 0.92–1.50) for coronary heart disease (CHD), 1.10 (95% CI 0.89–1.37) for stroke, and 1.30 (95% CI 1.07–1.56) for heart failure (HF). Decreased mtDNA-CN is linked to an increased risk of CVD and HF but not CHD and stroke. These findings suggest mtDNA-CN from leukocytes may be a potential early biomarker for CVD. However, more prospective studies with long follow-up are needed.

## Highlights

• Decreased mtDNA-CN is linked to higher risk of CVD and HF but not CHD or stroke
• More long-term studies are needed to link mtDNA-CN with CHD/stroke
• Leukocyte-derived mtDNA-CN may be an early biomarker for cardiovascular disease

## Graphical abstract

**Figure:**
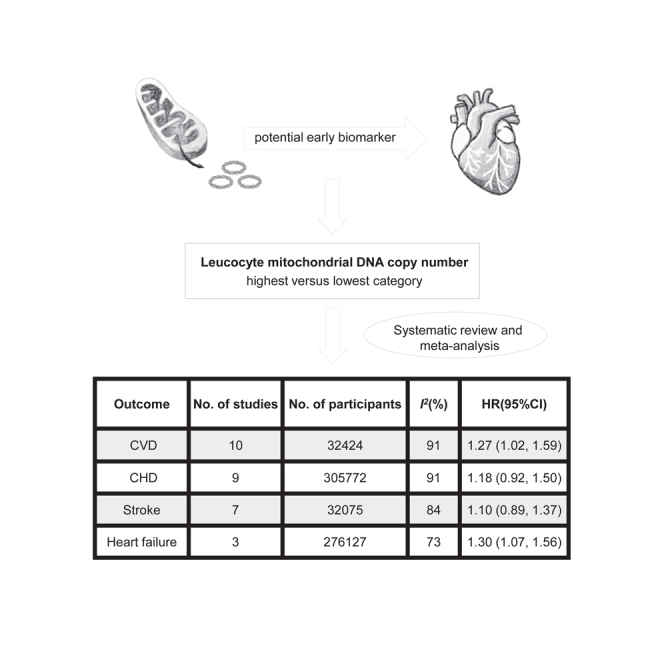

## Introduction

Cardiovascular disease (CVD) is the most prevalent non-communicable disease globally and a leading cause of death worldwide, accounting for 32% of all global deaths in 2019.^1^ Among CVD-related deaths, 85% were attributed to coronary heart disease (CHD) and stroke, resulting in significant health loss and posing a major barrier to sustainable human development.^1^^,^^2^^,^^3^^,^^4^ Therefore, gaining a deeper understanding of the factors that determine the development of CVD is crucial for early diagnosis prevention and intervention.

Mitochondria are double-membraned organelles primarily responsible for cellular metabolism and play crucial roles in various cellular processes, including aging, apoptosis, and oxidative phosphorylation.^5^^,^^6^ Dysfunction of mitochondria can impact disease susceptibility and severity.^7^ Mitochondrial DNA (mtDNA) is the only relatively independent genome within organelles, capable of controlling and encoding a portion of proteins and indirectly reflecting mitochondrial function.^8^ MtDNA change is associated with altered lipid metabolism, inflammatory response of resident arterial wall and circulating immune cells,^9^ and atherosclerosis development.^10^ Mitochondrial dysfunction is linked to increased oxidative stress,^11^ which is evidenced to be related to the pathology of CVD.^12^

Different from nuclear DNA, each mitochondrion contains between 2 and 10 copies of the mtDNA.^13^ Mitochondrial DNA copy number (mtDNA-CN) is a measure of mtDNA levels per cell and is considered a biomarker of mitochondrial function.^14^ The role of mtDNA-CN in the occurrence and development of CVD, as well as its rational application in treatment, will be important directions for future research.^8^ Experimental and epidemiological studies have shown that decreased levels of mtDNA-CN are related to the development of a series of chronic diseases, such as obesity,^15^^,^^16^ type 2 diabetes mellitus (T2DM),^15^^,^^16^^,^^17^^,^^18^ hypertension,^19^^,^^20^ atherosclerosis,^21^^,^^22^ which also suggest the potentially important role of mtDNA-CN in the development of CVD. In the past years, an increasing number of epidemiological studies also suggest that variations in mtDNA-CN may be associated with CVD and its subtypes including CHD, stroke, and heart failure (HF)^23^^,^^24^^,^^25^; however, the conclusions remain uncertain.^26^^,^^27^^,^^28^^,^^29^ Some studies showed a significant negative association of mtDNA-CN with CVD,^27^ CHD,^23^ stroke,^29^ and HF.^30^ Nevertheless, other studies did not find a significant association between mtDNA-CN and CVD,^31^ CHD,^26^ stroke,^27^ or HF.^28^ For example, Ashar et al.^23^ found that mtDNA-CN was independently associated with total CVD events in three large prospective studies, but in the adjusted model in two cohorts, there was no significant association found between mtDNA-CN and CHD or stroke. Although one meta-analysis by Peng Yue^32^ has explored the association between mtDNA-CN and CVD, the study included both cross-control and cohort studies and did not explore the association between mtDNA-CN and CHD, stroke, and HF. To our knowledge, no comprehensive systematic review or meta-analysis of cohort studies has been conducted to explore the association between mtDNA-CN and the risk of CVD. Moreover, much more cohort studies with large sample size^26^^,^^27^^,^^29^^,^^31^ have been performed on the association between mtDNA-CN and CVD and a systematic review and meta-analysis on the association of mtDNA-CN with CHD, stroke, and HF is lacking.

Therefore, this systematic review and meta-analysis of cohort studies sought to explore the association between mtDNA-CN and CVD, as well as CHD, stroke, and HF. This effort can help to bridge the research gap on mtDNA-CN and CVD and its subtypes and provide valuable insights for the early identification and diagnosis of CVD as potential new biomarkers.

## Results

### Study selection

**Figure 1:**
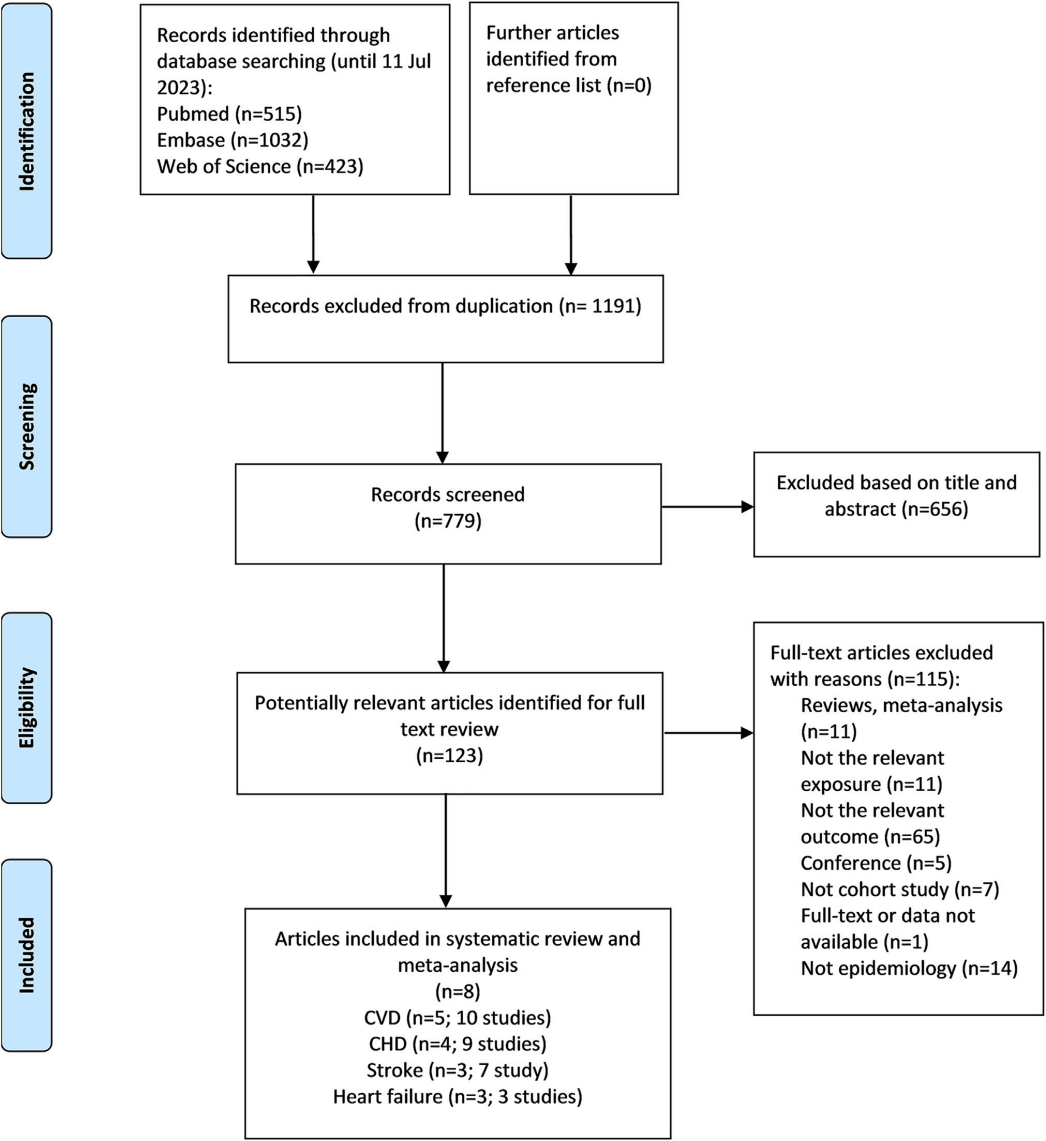
Flow chart of study selection

Flowchart of study selection is presented in Figure 1. Of a total of 1,970 articles identified through the search strategy, 779 articles remained after removing 1,191 duplicates. After reviewing the titles and abstracts, 656 articles were excluded based on our selection criteria. Following the full-text review of 123 articles, we identified and selected 8 eligible articles for our systematic review. Among these 8 articles, 4 articles contain multiple studies: Liu et al.^26^ included 11 studies involving four different cohorts (the Framingham Heart Study (FHS), Genetic Epidemiology Network of Arteriopathy (GENOA), Jackson Heart Study (JHS), and Women’s Health Initiative (WHI)) that studied CVD, four (FHS, GENOA, JHS, and WHI) that studied CHD, and three (FHS, JHS, and WHI) that studied stroke. Ashar et al.^23^ had 9 studies involving three different cohorts (the Atherosclerosis Risk in Communities Study (ARIC), Cardiovascular Health Study (CHS), and Multi-Ethnic Study of Atherosclerosis (MESA)) that studied CVD, CHD, and stroke. Sundquist et al.^27^ included 3 studies using the WHILA cohort to investigate CVD, CHD, and stroke. Luo et al.^29^ included 2 studies using the UK Biobank (UKB) cohort, which studied CHD and HF respectively. Thus, these 8 articles encompassed a total of 29 studies (5 articles with 10 studies for CVD,^23^^,^^26^^,^^27^^,^^33^ 4 articles with 9 studies for CHD,^23^^,^^26^^,^^27^^,^^29^ 3 articles with 7 studies for stroke,^23^^,^^26^^,^^27^ and 3 articles with 3 studies for HF^28^^,^^29^^,^^30^). These articles all provided a survival HR in their analysis and were therefore included in the meta-analysis.

### Study characteristics

Table 1 summarizes the main characteristics of the included studies. Of the 8 articles, the follow-up duration ranged from 2 to 24 years. The total number of participants included in this meta-analysis was 646,398, with 7,033 CVD cases, 22,500 CHD cases, 3,419 stroke cases, and 8,140 HF cases.

**Table 1:** Characteristics of studies included in the systematic review and meta-analysis

Study	Country	Data source (Sample size/cases)	Follow-up (y)	Proportion of women	Mean age	Source of mtDNA	Methods of mtDNA-CN assessment	Adjustments	CVD	CHD	Stroke	HF
Liu et al.^26^	USA	FHS: (CVD: 1703/189; CHD:1716/95; Stroke: 1816/69) GENOA: (CVD: 358/12; CHD: 372/10) JHS： (CVD: 2604/333; CHD: 2392/108; Stroke: 2511/99) WHI: (CVD: 4178/12; CHD: 4448/1250; Stroke: 4523/1558)	11	FHS: 68.00% GENOA: 68.00% JHS: 68.00% WHI: 100.00%	62	whole blood	WGS	age, sex, study center (if applicable), race/ethnicity, BMI, TC, HDL, SBP, HRX, current smoking, diabetes, WBC, NE and PLT	＋	＋	＋	–
Sundquist et al.^27^	Sweden	WHILA: (CVD:3062/360; CHD:3062/231; Sroke:3062/110)	17	100.00%	57.5	whole blood	droplet digital PCR	age, smoking, educational level and physical activity	＋	＋	＋	–
Sundquist et al.^27^	Sweden	WHILA (2508/118)	17	100.00%	57.5	whole blood	droplet digital PCR	age, smoking, educational level and physical activity	–	–	–	＋
Koller et al.^31^	UK	CAVASIC (236/35)	7	0	57.67	whole blood	Plasmid-normalized qPCR	age, current smoking, ln-CRP, diabetes mellitus, prevalent CVD, leukocytes and platelets	＋	–	–	–
Ashar et al.^23^	USA	ARIC: (CVD:10150/1500; CHD: 10150/994; Sroke:10150/634) CHS: (CVD:4126/1743; CHD: 4126/1197; Sroke:4126/780) MESA: (CVD:5887/422; CHD: 5887/269; Sroke:5887/169)	13.5	54.70%	ARIC: 57.9 CHS: 72.5 MESA: 62.4	ARIC: whole blood CHS: whole blood MESA: peripheral leukocytes	ARIC: Affymetrix Genome-Wide Human SNP Array 6.0 CHS: multiplexed TaqMan-based quantitative PCR MESA: Affymetrix Genome-Wide Human SNP Array 6.0	age, sex, collection center, race, total cholesterol, high-density lipoprotein cholesterol, systolic blood pressure, current smoking status, hypertension medication status, and type2 diabetes status	＋	＋	＋	–
Yoon et al.^33^	Korea	YUHS (120/16)	2.95	57.50%	52.3	peripheral whole blood	SYBR green-based quantitative PCR	age, sex, peritoneal dialysis duration, previous history of coronary artery disease, serum albumin, high-sensitivity C-reactive protein, and malnutrition inflammation score	＋	–	–	–
Luo et al.^29^	UK	UKB (273619/18346)	11.8	55.14%	54.5	leukocytes	intensities of genotyping probes on the mitochondrial chromosome on the Affymetrix Array	age, sex, genotyping batch, the first two principal components, white blood cell count, and platelet count, body mass index, physical activity, smoking status, alcohol consumption frequency, blood pressure and blood pressure-lowering medication, cholesterol, triglycerides and lipid-lowering medication, sleep duration and insomnia, type 2 diabetes status, and familial history of cardiovascular disease. CAD, coronary artery disease; HF, heart failure	–	＋	–	＋
Hong et al.^30^	USA	ARIC (10802/2227)	23.1	54.60%	55	peripheral blood	Affymetrix Genome-Wide Human SNP Array 6.0	age, sex, race/ethnicity, center, body mass index, smoking, alcohol intake, total and high-density lipoprotein cholesterol, cholesterol medication, hypertension, diabetes, and prevalent coronary heart disease	–	–	–	＋

BMI, body mass index; mtDNA, mitochondrial DNA; mtDNA-CN, mitochondrial DNA copy number; CVD, cardiovascular disease; CHD, coronary heart disease; HF, heart failure; CAD, coronary artery disease; PCR, polymerase chain reaction. WBC, White Blood Cell; NE, Neutrophil; PLT, Platelet; TC, total cholesterol; HDL, high-density lipoprotein cholesterol; SBP, systolic blood pressure, HRX, treatment for high blood pressure or hypertension; PCR, polymerase chain reaction; FHS, Framingham Heart Study; GENOA, Genetic Epidemiology Network of Arteriopathy Study; JHS, Jackson Heart Study; ARIC, Atherosclerosis Risk in Communities study; WHILA, Women health in Lund area; UKB, the UK Biobank; MESA, Multi-Ethnic Study of Atherosclerosis; CHS, Cardiovascular Health Study; WHI, Women’s Health initiative; WGS, whole genome sequencing; YUHS, Yonsei University Health System; CAVASIC, Cardiovascular Disease in Intermittent Claudication study.

All articles measured mtDNA-CN in blood, including both whole blood and peripheral leukocytes. Three primary methods were used for mtDNA-CN detection: whole genome sequencing (WGS) in 11 studies,^26^ the Affymetrix Genome-Wide Human SNP Array in 9 studies,^23^^,^^29^^,^^30^ and the remaining studies employed polymerase chain reaction (PCR) methods (4 using droplet digital PCR [ddPCR],^27^^,^^28^ 1 using plasmid-normalized quantitative PCR [qPCR],^31^ 3 using multiplexed TaqMan-based quantitative PCR,^23^ and 1 using SYBR green-qPCR^33^).

In the meta-analysis, 7 studies included only females from,^27^^,^^28^ 1 study only had males from the Cardiovascular Disease in Intermittent Claudication (CAVASIC) study,^31^ and the rest of the studies included both males and females.^26^^,^^29^^,^^30^^,^^33^ According to the NOS, all studies were assessed as moderate to high-quality research (Table S2).

### Mitochondrial DNA copy number and cardiovascular diseases

A total of 5 articles (10 studies)^23^^,^^26^^,^^27^^,^^31^^,^^33^ explored the relationship between mtDNA-CN and CVD. Most studies (7 studies) were conducted in the United States (US),^23^^,^^26^ one in Sweden,^27^ one in the United Kindom (UK),^29^ and one in Korea.^33^

Ashar et al.^23^ followed up with 21,870 participants from 3 separate cohorts for an average of 13.5 years, including White, Black, Hispanic, and Chinese individuals. They found that in all 3 cohorts, mtDNA-CN was negatively correlated with the incidence of CVD, demonstrating its potential as a clinically useful predictive factor for CVD. However, in the study conducted by Liu et al.,^26^ which only included data from 4 different studies due to overlap with Ashar et al.,^23^ no significant association was found between mtDNA-CN and CVD.

Additionally, in a prospective study conducted by Sundquist et al.^27^ in middle-aged Swedish women, it was found that lower mtDNA-CN was independently associated with future risk of CVD. Participants in the lowest quartile of mtDNA-CN had a 1.5 times higher risk of developing CVD compared to those in the highest quartile. Nevertheless, a study involving peritoneal dialysis patients did not find any evidence of a correlation between a decrease in mtDNA-CN and the incidence of CVD.^33^ Koller et al.^31^ obtained similar results, with no significant correlation found between mtDNA-CN in the lowest quartile and cardiovascular events in peripheral artery disease patients after adjustment.

**Figure 2:**
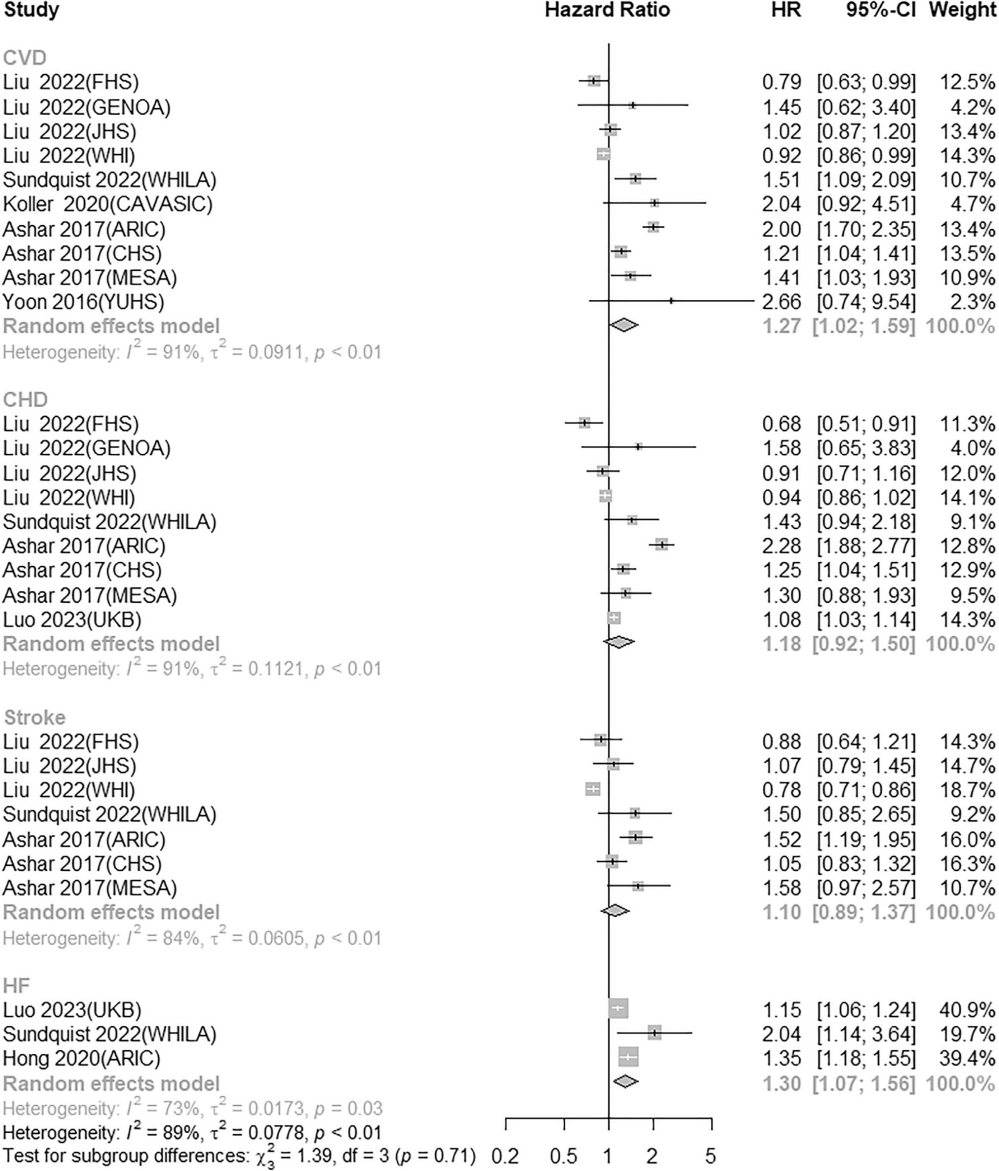
Forest plot for the pooled association between mitochondrial DNA copy number and risk of cardiovascular diseases for the highest versus lowest meta-analysis

Comparing the lowest quartile of mtDNA-CN to the highest quartile, an increased risk of CVD was observed (summary HR = 1.27; 95% CI: 1.02–1.59), with high heterogeneity across studies (*I*^*2*^ = 91%, *P*_heterogeneity_ <0.01) (Figure 2). Upon visual inspection, the funnel plot appears asymmetric (Figure S1A), but the results of Egger’s test are not statistically significant (*p* = 0.16).

In the subgroup analyses, mtDNA-CN exhibited a positive correlation in the subgroups that were conducted in Sweden, subgroups of age≤60, follow-up years>11, mtDNA sourced from leukocytes, studies adjusting for smoking, and without adjusting for BMI, studies with moderate quality, and in people with underlying diseases. However, moderate to high heterogeneity was observed in most subgroups. The results of univariate meta-regression analysis are presented in Table 2. Among the included covariates, we found that age, follow-up years, and adjustment for BMI could explain the high heterogeneity (*p* < 0.01).

**Table 2:** Subgroup analysis of mitochondrial DNA copy number and risk of CVD for the lowest versus highest meta-analysis

Subgroups	No. of studies	RR (95% CI)	*I*^*2*^*%*	*P*_*1*_^2^	*P*_*2*_^3^
**CVD**	10	1.27(1.02, 1.59)	91	<0.01	

**Country**					

USA	7	1.18(0.92, 1.52)	93	<0.01	0.30
Sweden	1	1.51(1.09, 2.09)	–	–	0.63
UK	1	2.04(0.92, 4.51)	–	–	Ref.
Korea	1	2.66(0.74, 9.54)	–	–	0.16

**Sex**					

Men & women	7	1.28(0.97, 1.69)	90	<0.01	Ref.
Women	2	1.15(0.71, 1.86)	88	<0.01	0.38
Men	1	2.04(0.92, 4.51)	–	–	0.70
Age					
≤60	4	1.86(1.52, 2.29)	0	0.46	<0.01
>60	6	1.04(0.88, 1.24)	75	<0.01	Ref.

**No of participants**					

≤4000	6	1.22(0.88, 1.69)	70	0.08	0.75
>4000	4	1.32(0.95, 1.84)	96	<0.01	Ref.

**No of cases**					

≤400	6	1.22(0.88, 1.69)	70	<0.01	0.75
>400	4	1.32(0.95, 1.84)	96	<0.01	Ref.

**Follow-up years**					

≤11	5	0.94(0.85, 1.03)	52	0.08	<0.01
>11	5	1.54(1.22, 1.94)	81	<0.01	Ref.

**Source of mtDNA**					

whole blood	9	1.26(0.98, 1.61)	92	<0.01	0.77
leukocytes	1	1.41(1.03, 1.93)	–	–	Ref.

**Adjustment for smoking**					

Yes	9	1.25(1.00, 1.56)	92	<0.01	0.30
No	1	2.66(0.74, 9.54)	–	–	Ref.

**Adjustment for BMI**					

Yes	4	0.93(0.86, 0.99)	33	0.21	<0.01
No	6	1.57(1.25, 1.95)	77	<0.01	Ref.

**Study quality**					

High	8	1.21(0.96, 1.52)	93	<0.01	0.15
Moderate	2	2.20(1.12, 4.31)	0	0.73	Ref.

**Disease status**					

Non-Diseased	8	1.21 (0.96, 1.52)	93	<0.01	0.15
Diseased	2	2.20 (1.12, 4.31)	0	0.73	Ref.

CI, confidence interval; RR, relative risk; BMI, body mass index; ***P***_***1***_: *p* value for heterogeneity within each subgroup; ***P***_***2***_***:****p* value for heterogeneity between subgroups with meta-regression analysis; mtDNA, mitochondrial DNA; CVD, cardiovascular disease.

Sensitivity analysis revealed that after excluding the studies conducted by Sundquist et al.,^27^ Koller et al.,^31^ and Ashar (ARIC) et al.,^23^ the association between mtDNA-CN and the risk of CVD became statistically insignificant.

### Mitochondrial DNA copy number and coronary heart disease

A total of 4 articles (9 studies)^23^^,^^26^^,^^27^^,^^29^ have explored the relationship between mtDNA-CN and CHD. Studies were mainly conducted in the US^23^^,^^26^ for seven of them, one study was conducted in Sweden,^27^ and one in the UK.^29^

Ashar et al.^23^ included 11,153 participants from ARIC, 4,830 from CHS, and 5,887 from MESA, for a total sample size of 21,870 individuals. The average age of the participants was 62.4 years. After adjusting for age, gender, race/ethnicity, and study center, the pooled HRs for incident CHD associated with a 1-SD decrease in mtDNA-CN was 1.29 (95% CI: 1.24–1.33), with a stronger association observed in the ARIC study. In Luo et al.'s study,^29^ adjusted HRs for the first (lowest mtDNA abundance) vs. the fifth (reference, highest mtDNA abundance) quintile were 1.08 (95% CI: 1.03–1.14) for coronary artery disease (CAD). However, Liu et al.^26^ obtained opposite results and did not find a significant association between mtDNA-CN and CHD in the adjustment model using the different cohorts. Sundquist et al.^27^ stratified data based on the type of CVD, and it revealed that lower baseline mtDNA-CN was not significantly associated with an increased risk of CHD (HR = 1.43; 95% CI: 0.94–2.18).

The meta-analysis, comparing the lowest quartile to the highest quartile of mtDNA-CN, resulted in a summary HR of 1.18 (95% CI: 0.92–1.50), with high heterogeneity (*I*^*2*^ = 91%; *P*_heterogeneity_ <0.01) (Figure 2). The funnel plot (Figure S1B) we obtained for qualitative assessment and Egger’s test (*p =* 0.53) for quantitative assessment did not reveal any publication bias. In subgroup analyses, there was an association between lower mtDNA-CN and higher risk of CHD in the subgroup with follow-up years>11, mtDNA sourced from leukocytes, and the subgroup without BMI adjustment. Meta-regression suggested that adjustment for BMI contributed to the high heterogeneity (*P*_heterogeneity_ <0.01). Sensitivity analysis indicated that after omitting the FHS cohort analysis conducted by Liu et al.,^26^ lower mtDNA-CN was associated with the risk of CHD (HR = 1.26, 95% CI: 1.00–1.59) (Table S3).

### Mitochondrial DNA copy number and stroke

A total of 3 articles (7 studies)^23^^,^^26^^,^^27^ have explored the relationship between mtDNA-CN and stroke. Studies were mainly conducted in the US for six of them,^23^^,^^26^ except that only one study was conducted in Sweden.^27^

Two studies^23^^,^^27^ were conducted in women (WHILA and WHI), while the remaining studies were conducted in the general population. Apart from Ashar et al.'s study^23^ from the ARIC study, all other studies showed that low mtDNA-CN was not significantly associated with incident stroke.

Figure 2 showed a summary HR of 1.10 (95% CI: 0.89–1.37) for stroke using the random-effects model when comparing the lowest quartile of mtDNA-CN to the highest quartile, with high heterogeneity (*I*^*2*^ = 84%; *P*_heterogeneity_ <0.01) indicated. The results of Egger’s test (*p* = 0.03) indicated potential publication bias in the meta-analysis and the trim and fill method showed consistent results (HR = 0.84; 95% CI: 0.63–1.11). In most subgroups, there was no significant association between mtDNA-CN and stroke risk (Table 3), and moderate to high heterogeneity was observed. To further investigate the high heterogeneity, we conducted univariate meta-regression analysis, which showed that follow-up years and adjustment for BMI may be sources of heterogeneity. Sensitivity analysis results suggest that HR values remain relatively stable in terms of direction and significance across outcomes (Table S3).

**Table 3:** Subgroup analysis of mitochondrial DNA copy number and risk of CHD, stroke, and HF for the lowest versus highest meta-analysis

Subgroups	No. of studies	RR (95% CI)	*I*^*2*^*%*	*P*_*1*_^2^	*P*_*2*_^3^
**CHD**	9	1.18(0.92, 1.50)	91	<0.01	

**Country**					

USA	7	1.17(0.85, 1.59)	93	<0.01	0.66
Sweden	1	1.43(0.94, 2.18)	–	–	Ref.
UK	1	1.08(0.92, 1.50)	–	–	0.63

**Sex**					

Men & women	7	1.28(0.97, 1.69)	90	<0.01	Ref.
Women	2	1.43(0.71, 1.86)	88	<0.01	0.85
Men	0	–	–	–	–

**Age**					

≤60	3	1.18(0.96, 2.40)	96	<0.01	0.08
>60	6	1.00(0.82, 1.23)	71	<0.01	Ref.

**No of participants**					

≤4000	4	1.00(0.69, 1.44)	69	0.02	0.31
>4000	5	1.29(0.95, 1.76)	94		Ref.

**No of cases**					

≤400	5	1.05(0.77, 1.43)	68	0.01	0.43
>400	4	1.29(0.89, 1.89)	96	<0.01	Ref.

**Follow-up years**					

≤11	4	0.89(0.76, 1.04)	49	0.12	0.02
>11	5	1.41(1.07, 1.87)	93	<0.01	Ref.

**Source of mtDNA**					

whole blood	7	1.18(0.96, 1.62)	93	<0.01	0.98
leukocytes	2	1.08(1.03, 1.14)	0	0.36	Ref.

**Adjustment for smoking**					

Yes	9	1.18(0.92, 1.50)	91	<0.01	0.19
No	0	–	–	–	–

**Adjustment for BMI**					

Yes	5	0.94(0.80, 1.10)	77	<0.01	<0.01
No	4	1.54(1.13, 2.10)	86	<0.01	Ref.

**Study quality**					

High	9	1.18(0.92, 1.50)	91	<0.01	0.19
Moderate	0	–	–	–	–
**Stroke**	7	1.10(0.89, 1.37)	84	<0.01	

**Country**					

USA	6	1.07(0.85, 1.35)	85	<0.01	0.40
Sweden	1	1.50(0.85, 2.65)	–	–	Ref.

**Sex**					

Men & women	6	1.28(0.97, 1.69)	90	<0.01	Ref.
Women	2	1.15(0.71, 1.86)	88	<0.01	0.35
Men	0	–	–	–	–

**Age**					

≤60	5	0.98(0.80, 1.20)	73	<0.01	0.03
>60	2	1.52(1.21, 1.90)	0	0.97	Ref.

**No of participants**					

≤4000	3	1.03(0.84, 1.28)	24	0.27	0.84
>4000	4	1.14(0.81, 1.60)	91	<0.01	Ref.

**No of cases**					

≤400	4	1.15(0.88, 1.49)	41	0.16	0.67
>400	3	1.06(0.72, 1.56)	93	<0.01	Ref.

**Follow-up years**					

≤11	3	0.86(0.71, 1.05)	51	0.13	<0.01
>11	4	1.34(1.05, 1.70)	47	0.13	Ref.

**Source of mtDNA**					

whole blood	6	1.06(0.85, 1.33)	84	<0.01	0.27
leukocytes	1	1.58(0.97, 2.57)	–	–	Ref.

**Adjustment for smoking**					

Yes	7	1.10(0.89, 1.37)	84	<0.01	0.37
No	0	–	–	–	–

**Adjustment for BMI**					

Yes	3	0.86(0.71, 1.05)	51	0.13	<0.01
No	4	1.34(1.05, 1.70)	47	0.13	Ref.

**Study quality**					

High	7	1.10(0.89, 1.37)	84	<0.01	0.37
Moderate	0	–	–	–	–
**HF**	3	1.30(1.07, 1.56)	73	0.03	

**Country**					

USA	1	1.35(1.18, 1.55)	–	–	
Sweden	1	2.04(1.14, 3.64)	–	–	
UK	1	1.15(1.06, 1.24)	–	–	

**Sex**					

Men & women	2	1.23(1.06, 1.44)	76	0.04	Ref.
Women	1	2.04(1.14, 3.64)	–	–	0.12
Men	0	–	–	–	

**Age**					

≤60	3	1.30(1.07, 1.56)	73	0.03	
>60	0	–	–	–	

**No of participants**					

≤4000	2	1.23(1.06, 1.44)	76	0.04	0.12
>4000	1	2.04(1.14, 3.64)	–	–	Ref.

**No of cases**					

≤400	2	1.23(1.06, 1.44)	76	0.04	0.12
>400	1	2.04(1.14, 3.64)	–	–	Ref.

**Follow-up years**					

≤11	3	1.30(1.07, 1.56)	73	0.03	
>11	0	–	–	–	

**Source of mtDNA**					

whole blood	1	1.50(1.06, 2.14)	46	0.17	0.32
leukocytes	2	1.15(1.06, 1.24)	–	–	Ref.

**Adjustment for smoking**					

Yes	3	1.30(1.07, 1.56)	73	0.03	
No	0	–	–	–	

**Adjustment for BMI**					

Yes	3	1.30(1.07, 1.56)	73	0.03	
No	0	–	–	–	

**Study quality**					

High	3	1.30(1.07, 1.56)	73	0.03	
Moderate	0	–	–	–	

CI, confidence interval; RR, relative risk; BMI, body mass index; ***P***_***1***_: *p* value for heterogeneity within each subgroup; ***P***_***2***_***:****p* value for heterogeneity between subgroups with meta-regression analysis; mtDNA, mitochondrial DNA; CHD, coronary heart disease; HF, heart failure.

### Mitochondrial DNA copy number and heart failure

A total of 3 articles (3 studies)^28^^,^^29^^,^^30^ have investigated the relationship between mtDNA-CN and HF, with one study conducted in the US,^30^ one in Sweden,^28^ and one in the UK.^29^

All 3 studies reported a significant association between lower mtDNA-CN and higher risk of HF. One recent study that was conducted in the UK, including 10,802 participants and 2,227 HF cases, indicated a negative correlation between mtDNA-CN and the risk of HF.^30^ Another study in Sweden including 2,508 participants and 118 HF cases demonstrated that for each 1-SD deviation decrease in baseline mtDNA-CN, the risk of incident HF increased by 34% (HR = 1.34; 95% CI: 1.11–1.63). Similar results were obtained when comparing the lowest quartiles of mtDNA-CN levels to the highest quartiles, showing a higher risk of HF incidence (HR = 2.04, 95% CI: 1.14–3.64).^28^ Luo et al.^29^ found that the adjusted HR for the lowest quintile of mtDNA abundance in relation to HF was 1.17 (95% CI: 1.05–1.23) compared to the highest quintile (reference), using the UK biobank involving 273,619 participants aged between 40 and 69 years.

The random-effects model showed that low mtDNA-CN was associated with a higher risk of developing HF (summary HR = 1.30; 95% CI: 1.07–1.56) (Figure 2), with high heterogeneity (*I*^*2*^ = 73%; *P*_heterogeneity_ <0.01) (Figure 2). The Egger’s test (*p* = 0.28) indicated no significant publication bias. Table 3 summarizes the combined effect sizes of subgroups defined by different study characteristics. In all subgroups, lower mtDNA-CN remained significantly associated with higher HF risk. In the meta-regression analysis, none of them yielded statistically significant results. Sensitivity analysis suggests stable findings, except that no significant association between mtDNA-CN and HF was found after excluding the study conducted by Hong et al..^30^

### Grading of the evidence

Table S4 provides the GRADE assessment of the evidence regarding the association between mtDNA-CN and CVD, CHD, stroke, and HF. The evidence quality for all outcomes is rated as very low. This is primarily due to a high risk of bias, low precision, and publication bias.

## Discussion

Using a comprehensive systematic review and meta-analysis of cohort studies, this study investigated the associations of mtDNA-CN with CVD and different subtypes of CVD including CHD, stroke, and HF. The results showed that lower mtDNA-CN in whole blood or peripheral leukocytes was associated with an increased risk of CVD. As for the subtypes of CVD, a significant association between lower mtDNA-CN levels and higher risk of HF was identified when comparing the lowest with the highest quartile of mtDNA-CN, whereas a non-significant association was found for both CHD and stroke.

It remains controversial whether mtDNA-CN is a potential predictor of CVD and its different subtypes. Despite of increasing number of prospective cohort studies, inconsistent findings have been reported, which stress the importance of performing a systematic review and meta-analysis to help researchers better understand the relationship between mtDNA-CN and CVD. One previous study by Peng Yue et al.^32^ conducted a meta-analysis on mtDNA-CN and CVD by including 5 articles (7 studies) with 8,252 cases published up to October 2017, of which only 4 included studies were cohort studies in the previous study. They found a significant negative correlation between mtDNA-CN and CVD risk, which was consistent with the results in the present study. Compared with the meta-analysis by Peng Yue et al.,^32^ our meta-analysis only included cohort studies to explore the association of mtDNA-CN and risk of CVD and firstly investigate the association between mtDNA-CN and CHD, stroke, and HF. Furthermore, we added more than 7 cohort studies to increase the reliability of the meta-analysis’s findings and enable us to perform subgroup analyses. Our study also investigated other CVD-specific outcomes including CHD, stroke, and HF and indicated a significant negative association was found for HF but not CHD and stroke.

High heterogeneity was found for all outcomes. For CVD and stroke, age, duration of follow-up, and adjustment for BMI were found to be potential sources of high heterogeneity as shown in the meta-regression analysis. Meta-regression analysis in the subgroup analyses of CHD showed that follow-up duration and adjustment for BMI are potential sources of heterogeneity.

Yoon et al.^33^ and Koller et al.^31^ have shown that after adjusting the models, there was no significant correlation between mtDNA-CN and CVD in populations undergoing peritoneal dialysis and those with peripheral artery disease. However, in our subgroup analyses for populations with and without underlying diseases, we found that lower mtDNA-CN was significantly associated with cardiovascular diseases in populations with underlying diseases but not in the general population. This might be because the random effects model adjusts the estimates of effect sizes based on the weight of each study, and combining data from multiple studies increases the sample size, thus enhancing the ability to detect actual effects.^34^ Although mitochondrial dysfunction may be prevalent among patients with CVD, the relationship between CVD risk and mitochondrial dysfunction remains elusive. A recent Mendelian randomization study found no causal relationship between genetically predicted mtDNA copy number and any cardiac metabolic diseases.^35^ Therefore, the variability in mtDNA-CN and its impact on CVD risk may be influenced by a multitude of pathological states or confounding factors. It is crucial to consider each patient’s specific disease background and other potential risk factors comprehensively when evaluating the relationship between mtDNA-CN and CVD risk.

It is noteworthy that we found an increased risk of CHD and stroke associated with lower mtDNA-CN in the subgroups of studies with follow-up larger than 11 years (5 cohort studies included for CHD and 4 for stroke), which suggests the change of mtDNA-CN may be an early biomarker in the long-term development of CVD and future more studies are needed to have a long follow up duration to explore the role in the mtDNA-CN in CVD, especially CHD and stroke. However, we cannot rule out the possibility that these differences may be due to the measurement methods of mtDNA-CN and sample sizes. Notably, studies with a follow-up duration of less than 11 years were all derived from Liu et al.’s^26^ research, which had relatively smaller sample sizes from cohorts such as FHS, GENOA, JHS, and WHI, and employed WGS to measure mtDNA-CN. Unlike traditional methods such as qPCR or ddPCR that target specific regions of the mitochondrial genome, WGS provides a more comprehensive genomic perspective. It is capable of detecting subtle variations across the entire mitochondrial genome, which may lead to more accurate and varied measurements of mtDNA-CN due to its extensive coverage.^36^^,^^37^

Although it remains unclear for the mechanism of the role of mtDNA-CN in the development of CVD, various aspects encompassing immune dysfunction, oxidative stress, inflammation, and altered cell signaling may help explain the mechanisms connecting mitochondrial dysfunction to CVD.^5^ First, changes in mitochondrial oxidative capacity may lead to chronic inflammation by affecting macrophage polarization^38^ and inflammation was found to occur from initiation through progression to eventual thrombotic events for CVD.^39^ Second, mtDNA damage can promote atherosclerosis and plaque vulnerability.^40^^,^^41^ Animal experiments have shown that mtDNA damage is an early event in the development of atherosclerosis.^21^ Some studies suggest that reduced mtDNA-CN levels are related to increased oxidative stress, leading to elevated production of reactive oxygen species (ROS).^42^ However, other research indicates that mitochondrial contribution to atherosclerosis is not linked to ROS production.^43^ Dysfunction in mtDNA can result in decreased expression of respiratory complexes and diminished mitochondrial respiration in vascular smooth muscle cells, monocytes/macrophages, and other organs. Decreased ATP levels can promote cell apoptosis, inhibit cell proliferation, and ultimately lead to atherosclerosis and plaque rupture.^21^^,^^44^ Furthermore, mitochondrial dysfunction can also impact nuclear gene expression and methylation patterns.^45^ Modifications in mtDNA-CN can result in changes in nuclear DNA methylation, thereby affecting the nuclear DNA expression of nearby genes and contributing to the pathological changes seen in CVD.^6^

MtDNA-CN holds potential significance as a biomarker for CVD, with its variations preceding both structural and functional changes in the heart, surpassing other clinical biomarkers such as BNP and NT-proBNP.^32^ It enables the early detection of CVD and timely intervention to prevent disease progression. Additionally, using mtDNA-CN to detect CVD is convenient and cost-effective for patients, as it requires only a small amount of peripheral blood. However, there are numerous methods for determining mtDNA-CN, and discrepancies may exist between different laboratories. Therefore, standardized procedures and criteria are needed to ensure the comparability and accuracy of results. Furthermore, to translate research findings into clinically prognostic interpretations, large-scale prospective studies are required to verify the causal relationship between mtDNA-CN and CVD. This includes determining specific mtDNA-CN thresholds, assessing its utility in various populations, and distinguishing patients at different risk levels to ensure its broad clinical applicability.

### Limitations of the study

The present systematic review and meta-analysis is the first to use a comprehensive systematic search of cohort studies to explore the association between mtDNA-CN and the risk of CVD as well as different CVD subtypes including CHD, stroke, and HF. However, several limitations in our study should be considered. First, a high degree of heterogeneity was observed in the meta-analysis, but we have performed a series of subgroup analyses and meta-regression to find the source of heterogeneity. Second, the subgroup analyses on the association of mtDNA-CN and CVD, CHD, and stroke were not robust, and limited studies focused on the main subtypes of CVD, especially HF, which suggests future more studies remain needed. Third, the quality of articles included in the study was limited, potentially introducing selection and information bias. Additionally, due to observational design, the overall evidence quality assessed by GRADE was very low, necessitating intervention studies to confirm this association. Fourth, despite the use of the most adjusted risk estimates, unaccounted confounding factors could still impact the strength of the relationship. Moreover, publication bias was detected in the meta-analysis on stroke, highlighting the scarcity of publications involving small sample sizes and studies that did not yield conclusive results. However, the trim and fill method showed stable results. Fifth, the statistical power of funnel plots and Egger’s test was limited due to the small number of studies for stroke and heart failure. The Cochrane Handbook^46^ suggests a minimum of 10 studies for these analyses to effectively distinguish chance from real asymmetry. Our analysis, with fewer studies, may thus have reduced sensitivity in detecting publication bias. Therefore, more studies are warranted to explore the association between mtDNA-CN and stroke and HF. Sixth, some subgroups were entirely based on data from a single study, which may limit the generalizability and reliability of these subgroup conclusions, as they may not apply to a broader population or other research settings. Moreover, when the data in a subgroup come solely from one study, the statistical power of the analysis may be insufficient, making it difficult to capture more subtle effects or to exclude the influence of random factors. Seventh, although most (2 articles, 7 studies) definitions of CVD include HF, stroke, and CHD, the definitions of CVD may vary between studies, which can impact the synthesis and interpretation of data in meta-analyses, as well as differences in study population sizes. Therefore, future studies should aim for a standardized definition of cardiovascular diseases to minimize variability across studies.

### Conclusions

In conclusion, we found that lower mtDNA-CN is associated with an increased risk of CVD and HF but not significantly associated with the risk of CHD and stroke. However, the data obtained in this study also suggest the need for further research, including studies with new mechanisms and cohort designs with long follow-up and different measurements of mtDNA-CN, to elucidate the biological effects of mtDNA-CN in CVD and its potential as a biomarker for predicting CVD.

## STAR★Methods

### Key resources table

**Table:** 

REAGENT or RESOURCE	SOURCE	IDENTIFIER
**Deposited data**		

Studies For Meta-analysis	PubMed, Web of Science database, and Embase	N/A

**Software and algorithms**		

R 4.3.1	R project	https://www.r-project.org/
RStudio 2023.09.0 + 463	RStudio	https://posit.co/download/rstudio-desktop/

### Resource availability

#### Lead contact

Further information and requests for resources and reagents should be directed to and will be fulfilled by the lead contact, Pei Qin (qinpei225@163.com).

#### Materials availability

This study did not generate unique reagents.

#### Data and code availability

• The data used in this meta-analysis came from published studies, and no new data or codes were used.
• All data are described in the ‘‘key resources table’’ section.
• Any additional information required to reanalyze the data reported in this paper is available from the lead contact upon request.

### Experimental model and study participant details

Our study does not use experimental models typical in the life sciences.

### Method details

#### Search strategy

We systematically searched the PubMed, Embase, and Web of Science databases, covering articles published from January 1, 1991 to July 11, 2023. The specific search terms are provided in Table S1. In addition, we meticulously reviewed the reference lists of extracted papers and recent reviews to identify any potentially omitted articles.

#### Eligibility criteria

Studies that met the following criteria were considered eligible for this meta-analysis: (1) the study population was general adults; (2) the exposure was mtDNA-CN and the outcomes included CVD, CHD, stroke, or HF; (3) the analysis involved comparing groups with higher levels versus lower levels of mtDNA-CN. (4) all data were presented as Odds Ratio (OR), Relative Risk (RR), Hazard Ratio (HR) and their 95% Confidence Intervals (CIs). (5) studies were performed in human beings and published in English. (6) they were cohort study design.

Publications were excluded if they met the following criteria: (1) reference abstracts, review articles, letters, comments, correspondence, and conference reports; (2) studies that did not provide enough data. All eligible articles for further review were initially screened based on their titles or abstracts, followed by a full-text review. If duplicate data sources were reported, we only included the study with the largest sample size for the meta-analysis.

#### Data extraction

Two researchers (X.L. and P.Q.) independently searched, screened the literature, and extracted the following data from the retrieved articles by using the inclusion and exclusion criteria: the first author’s last name, publication year, country, follow-up years, proportion of women, age of participants, sample size, number of cases, source of mtDNA, methods of mtDNA-CN assessment, definition and assessment methods of interested outcomes (CVD, CHD, stroke, and HF), full adjusted HRs and 95% CIs for the association, and adjusted variables. Study quality was assessed by the Newcastle–Ottawa Quality Assessment Scale (NOS) to evaluate three quality parameters (selection, comparability, and outcome) divided across nine specific items, and studies with scores of 0–3, 4 to 6, and 7 to 9 were considered low, moderate, and high quality.^47^

#### Grading of the evidence

The certainty of the evidence was appraised using the Grading of Recommendations Assessment, Development and Evaluation (GRADE) approach.^48^ The quality of evidence for each outcome was rated as high, moderate, low, or very low. The strength of observational studies was first rated as low-quality evidence and then upgraded or downgraded based on risk of bias, imprecision, indirectness, inconsistency, publication bias, and other considerations.

### Quantification and statistical analysis

Summary HRs with 95% CIs comparing the lowest to the highest levels of mtDNA-CN were calculated using a random-effects model, which is a more appropriate model to capturing uncertainty resulting from heterogeneity among studies [37, 38]. Heterogeneity was assessed using *I*^*2*^ values.^49^ The results of this step were illustrated with a forest plot. Publication bias was assessed by the funnel plot and Egger’s test.^50^ Sensitivity analysis, subgroup analyses, and meta regression, were performed to figure out the major factors of high heterogeneity. All tests were 2-sided and *p* < 0.05 was considered statistically significant. Statistical analyses were conducted using R 4.3.1.

### Additional resources

This systematic review and meta-analysis was registered in PROSPERO (CRD42023456231) and strictly followed the Preferred Reporting Items for Systematic reviews and Meta-Analyses (PRISMA) statement.^51^
