# Supplementary material for: Leukocyte mitochondrial DNA copy number and cardiovascular disease: A systematic review and meta-analysis of cohort studies
Source: iScience. 2024 Jul 17;27(9):110522. doi: 10.1016/j.isci.2024.110522 (PMC11363494; doi:10.1016/j.isci.2024.110522)
Supplement: Document S1. Figure S1 and Tables S1–S4 [file mmc1.pdf]

iScience, Volume *27*

Supplemental information

Leukocyte mitochondrial DNA copy number and cardiovascular disease: A systematic review and meta-analysis of cohort studies

Xinying Li, Xiaoning Liu, Xiaojuan Chen, Yanqi Wang, Shuning Wu, Fengjuan Li, Yuhao Su, Lifang Chen, Jian Xiao, Jianping Ma, and Pei Qin

Supplementary Material

Leucocyte mitochondrial DNA copy number and cardiovascular disease: a systematic review and meta-analysis of cohort studies

Table S1. Systematic literature review search terms and strategy, related to STAR Methods.

Table S2. Quality assessment of included prospective studies for the association between Mitochondrial copy number and cardiovascular diseases, related to STAR Methods.

Table S3. Sensitivity analysis of mitochondrial DNA copy number and risk of cardiovascular diseases for the highest versus lowest meta-analysis, related to STAR Methods.

Table S4. GRADE evidence profile for observational studies of mitochondrial DNA copy number and cardiovascular diseases, related to STAR Methods.

Figure S1. Funnel plots for the detection of publication bias of included studies for highest vs. lowest mitochondrial DNA copy number quartile and risk of cardiovascular diseases, related to STAR Methods. (A) CVD; (B) CHD.

Table S1. Systematic literature review search terms and strategy, related to STAR Methods.

**Search terms for PubMed (n=515), until 11 Jul 2023**
#1 ("Copy Number"[Title/Abstract] OR "content"[Title/Abstract]) AND ("DNA, Mitochondrial"[Mesh] OR "mtDNA"[Title/Abstract] OR "Mitochondrial DNA"[Title/Abstract])
#2 ("cerebrovascular disorders" [Mesh] OR "Cardiovascular Diseases"[Mesh] OR "cerebrovascular disorders" [Title/Abstract] OR "cardiovascular disease" [Title/Abstract] OR "cardiovascular diseases" [Title/Abstract] OR "CVD" [Title/Abstract] OR "coronary disease" [Title/Abstract] OR "coronary artery disease" [Title/Abstract] OR "coronary heart disease" [Title/Abstract] OR "CHD" [Title/Abstract] OR "ischemic heart disease" [Title/Abstract] OR "ischaemic heart disease" [Title/Abstract] OR "stroke" [Title/Abstract] OR "cerebrovascular disease" [Title/Abstract] OR "cerebrovascular disorders" [Title/Abstract] OR "heart disease" [Title/Abstract] OR "myocardial infarction" [Title/Abstract] OR "MI" [Title/Abstract] OR "heart failure" [Title/Abstract] OR "cerebral vascular accident" [Title/Abstract] OR "CVA" [Title/Abstract] OR "cardiovascular" [Title/Abstract] OR "coronary" [Title/Abstract] OR "myocardial" [Title/Abstract])
#1 AND #2
**Search terms for Embase (n=1032), until 11 Jul 2023**
#1 'mitochondrial DNA'/exp AND ('copy number'/exp OR 'content')
#2 cerebrovascular disorders OR (cerebrovascular disease or cardiovascular diseases) or (cardiovascular disease or coronary heart disease) or heart disease or heart disease or ischemic heart disease or ischaemic heart disease or ischemic heart disease or ischemic heart disease or coronary artery disease or CHD or myocardial infarction or heart infarction or stroke or cerebrovascular accident or ischemic stroke or brain ischemia or haemorrhagic stroke or brain hemorrhage or hemorrhagic stroke or brain hemorrhage or CVD or coronary disease or heart failure or heart failure or cerebral vascular accident or cerebrovascular accident or cardiovascular or coronary or myocardial
#1 AND #2
**Search terms for Web of Science (n=423), until 11 Jul 2023**
TS=(("Copy Number" OR "content") AND ("DNA, Mitochondrial" OR "mtDNA" OR "Mitochondrial DNA")) AND ("cerebrovascular disorders" OR "cerebrovascular disease" OR "cardiovascular disease" OR "cardiovascular disease" OR "coronary heart disease" OR "heart disease" OR "heart disease" OR "ischemic heart disease" OR "ischaemic heart disease" OR "ischemic heart disease" OR "ischemic heart disease" OR "coronary artery disease" OR "CHD" OR "myocardial infarction" OR "heart infarction" OR stroke OR "cerebrovascular accident" OR "ischemic stroke" OR "brain ischemia" OR "haemorrhagic stroke" OR "brain hemorrhage" OR "hemorrhagic stroke" OR "brain hemorrhage" OR "CVD" OR "coronary disease" OR "heart failure" OR "heart failure" OR "cerebral vascular accident" OR "cerebrovascular accident")

Table S2. Quality assessment of included prospective studies for the association between Mitochondrial copy number and cardiovascular diseases[1], related to STAR Methods.

First author (year)	Study Selection				Comparability of cohorts		Outcome			Total
	a	b	c	d	e	f	g	h	i	
**CVD**										
Liu 2022(FHS)	0	1	1	1	1	1	1	1	0	7
Liu 2022(GENOA)	1	1	1	1	1	1	1	1	1	9
Liu 2022(JHS)	1	1	1	1	1	1	1	1	1	9
Liu 2022(WHI)	0	1	1	1	1	1	1	1	1	8
Sundquist 2022(WHILA)	1	1	1	1	1	1	1	1	1	9
Koller 2020(CAVASIC)	0	1	1	1	1	1	1	0	0	6
Ashar 2017(ARIC)	1	1	1	1	1	1	1	1	1	9
Ashar 2017(CHS)	1	1	1	1	1	1	1	1	1	9
Ashar 2017(MESA)	1	1	1	1	1	1	1	1	1	9
Yoon 2016(YUHS)	0	0	1	1	1	0	1	1	0	5
**CHD**										
Liu 2022(FHS)	1	1	1	1	1	1	1	1	1	9
Liu 2022(GENOA)	1	1	1	1	1	1	1	1	1	9
Liu 2022(JHS)	1	1	1	1	1	1	1	1	1	9
Liu 2022(WHI)	0	1	1	1	1	1	1	1	1	8
Sundquist 2022(WHILA)	0	1	1	1	1	1	1	1	0	7
Ashar 2017(ARIC)	1	1	1	1	1	1	1	1	1	9
Ashar 2017(CHS)	1	1	1	1	1	1	1	1	1	9
Ashar 2017(MESA)	1	1	1	1	1	1	1	1	1	9
Luo 2023(UKB)	1	1	1	1	1	1	1	1	1	9
**Stroke**										
Liu 2022(FHS)	1	1	1	1	1	1	1	1	1	9
Liu 2022(JHS)	1	1	1	1	1	1	1	1	1	9
Liu 2022(WHI)	0	1	1	1	1	1	1	1	1	8
Sundquist 2022(WHILA)	0	1	1	1	1	1	1	1	0	7
Ashar 2017(ARIC)	1	1	1	1	1	1	1	1	1	9
Ashar 2017(CHS)	1	1	1	1	1	1	1	1	1	9
Ashar 2017(MESA)	1	1	1	1	1	1	1	1	1	9
**HF**										
Luo 2023(UKB)	0	1	1	1	1	1	1	1	0	7
Sundquist 2022(WHILA)	1	1	1	1	1	1	1	1	1	9
Hong 2020(ARIC)	1	1	1	1	1	1	1	1	1	9

[1]CVD, cardiovascular diseases; CHD, coronary heart diseases; HF, heart failure; FHS, Framingham Heart Study; GENOA, Genetic Epidemiology Network of Arteriopathy Study; JHS, Jackson Heart Study; ARIC, Atherosclerosis Risk in Communities study; WHILA, Women health in Lund area; UKB, the UK Biobank; MESA, Multi-Ethnic Study of Atherosclerosis; CHS, Cardiovascular Health Study; WHI, Women's Health initiative; YUHS, Yonsei University Health System; CAVASIC, Cardiovascular Disease in Intermittent Claudication study.

a. Representativeness of the exposed cohort;

b. Selection of the non-exposed cohort;

c. Ascertainment of exposure;
d. Demonstration that outcome of interest was not present at start of study;
e. Comparability of cohorts on the basis of the design or analysis (adjusted for age);
f. Comparability of cohorts on the basis of the design or analysis (adjusted for sex);
g. Assessment of outcome;
h. Was follow-up long enough for outcomes to occur;
i. Adequacy of follow-up of cohorts.

Table S3. Sensitivity analysis of mitochondrial DNA copy number and risk of cardiovascular diseases for the highest versus lowest meta-analysis [1], related to STAR Methods.

Study omitted	HR	95% CI	
**CVD**			
Liu 2022(FHS)	1.35	1.08	1.68
Liu 2022(GENOA)	1.26	1.00	1.60
Liu 2022(JHS)	1.32	1.03	1.69
Liu 2022(WHI)	1.34	1.05	1.70
Sundquist 2022(WHILA)	1.25	0.98	1.59
Koller 2020(CAVASIC)	1.24	0.99	1.55
Ashar 2017(ARIC)	1.15	0.95	1.38
Ashar 2017(CHS)	1.29	1.00	1.66
Ashar 2017(MESA)	1.26	0.98	1.61
Yoon 2016(YUHS)	1.25	1.00	1.56
**CHD**			
Liu 2022(FHS)	1.26	1.00	1.59
Liu 2022(GENOA)	1.16	0.90	1.49
Liu 2022(JHS)	1.22	0.93	1.59
Liu 2022(WHI)	1.22	0.93	1.60
Sundquist 2022(WHILA)	1.15	0.88	1.50
Ashar 2017(ARIC)	1.05	0.89	1.22
Ashar 2017(CHS)	1.17	0.88	1.54
Ashar 2017(MESA)	1.16	0.89	1.52
Luo 2023(UKB)	1.19	0.90	1.58
**Stroke**			
Liu 2022(FHS)	1.15	0.90	1.47
Liu 2022(JHS)	1.12	0.87	1.45
Liu 2022(WHI)	1.19	0.98	1.45
Sundquist 2022(WHILA)	1.07	0.85	1.35
Ashar 2017(ARIC)	1.02	0.83	1.26
Ashar 2017(CHS)	1.13	0.87	1.46
Ashar 2017(MESA)	1.06	0.85	1.33
**HF**			
Luo 2023(UKB)	1.50	1.06	2.14
Sundquist 2022(WHILA)	1.23	1.06	1.44
Hong 2020(ARIC)	1.42	0.83	2.44

[1] CI, confidence interval; RR, relative risk; BMI, body mass index; CVD, cardiovascular diseases; CHD, coronary heart diseases; HF, heart failure; FHS, Framingham Heart Study; GENOA, Genetic Epidemiology Network of Arteriopathy Study; JHS, Jackson Heart Study; ARIC, Atherosclerosis Risk in Communities study; WHILA, Women health in Lund area; UKB, the UK Biobank; MESA, Multi-Ethnic Study of Atherosclerosis; CHS, Cardiovascular Health Study; WHI, Women's Health initiative; YUHS, Yonsei University Health System; CAVASIC, Cardiovascular Disease in Intermittent Claudication study.

Table S4. GRADE evidence profile for observational studies of mtDNA copy number and cardiovascular diseases[1], Related to STAR Methods, related to STAR Methods.

Outcome	No of studies	Study design	Risk of bias	Inconsistency	Indirectness	Imprecision	Other considerations	Relative risk (95% CI)	Certainty
**CVD**	10	Observational studies	Not serious	Not serious	Not serious	Serious[b]	None	1.27(1.02, 1.59)	⊕◯◯◯ Very low
**CHD**	9	Observational studies	Not serious	Not serious	Not serious	Serious[c]	None	1.18(0.92, 1.50)	⊕◯◯◯ Very low
**Stroke**	7	Observational studies	Not serious	Not serious	Not serious	Serious[d]	Publication bias	1.10(0.89,1.37)	⊕◯◯◯ Very low
**HF**	3	Observational studies	Serious[a]	Not serious	Not serious	Not serious	None	1.30(1.07, 1.56)	⊕◯◯◯ Very low

[1] CI, confidence interval; CVD, cardiovascular diseases; CHD, coronary heart diseases; HF, heart failure.

a Serious inconsistency for HF due to high degree of unexplained heterogeneity.

b Serious imprecision for CVD, as the 95%CI (1.02-1.59) overlapped with the minimally important difference for clinical benefit (RR 1.05).

c Serious imprecision for CHD, as the 95%CI (0.92-1.50) overlapped with the minimally important difference for clinical harm (RR 1.05).

d Serious imprecision for stroke, as the 95%CI (0.89-1.37) overlapped with the minimally important difference for clinical harm (RR 1.05).

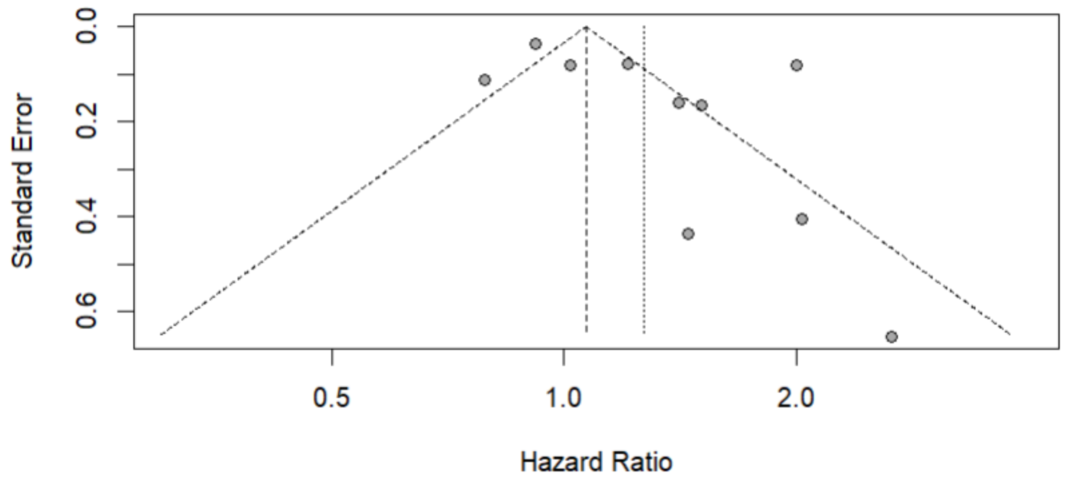

(A)

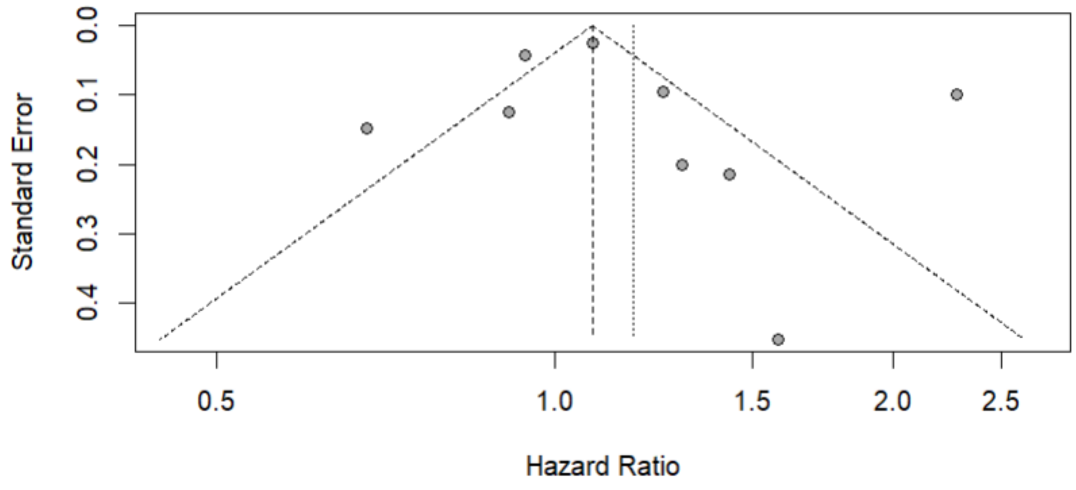

(B)

Figure S1. Funnel plots for the detection of publication bias of included studies for highest vs. lowest mitochondrial DNA copy number quartile and risk of cardiovascular diseases[1], related to STAR Methods. A.) CVD. B.) CHD.

[1]CVD, cardiovascular diseases; CHD, coronary heart diseases.
